# 1132. Evaluating an Amoxicillin Dosing Regimen for Community Acquired Pneumonia: A Quality Improvement Initiative Using Clinical and Laboratory Data

**DOI:** 10.1093/ofid/ofab466.1325

**Published:** 2021-12-04

**Authors:** Emma Keeler, Jonathan Beus, Molly Hayes, Joseph Zorc, Talene Metjian, Brandon Ku, Rose Hamershock, Jeffrey Gerber, Kathleen Chiotos

**Affiliations:** Children's Hospital of Philadelphia, Philadelphia, Pennsylvania

## Abstract

**Background:**

Amoxicillin 90 mg/kg/day divided twice daily is recommended for children with mild community acquired pneumonia (CAP). While adequate for fully susceptible *Streptococcus pneumoniae* isolates, three times daily dosing allows achievement of greater amoxicillin exposure, which may be necessary for isolates with penicillin minimum inhibitory concentrations (MIC) of ≥ 2 μg/mL. We evaluated our current twice daily amoxicillin dosing strategy by characterizing 1) the MIC distribution among *S. pneumoniae* isolates and 2) the frequency of clinical amoxicillin treatment failures.

**Methods:**

We performed a retrospective cohort study of all *S. pneumoniae* isolates from sterile and non-sterile sites between 2017-2020. Breakpoints established by the CLSI were used for both meningitis and non-meningitis isolates. Only the first isolate per patient was included. We also evaluated the frequency of amoxicillin treatment failure in patients diagnosed with CAP who were discharged from the ED in 2019. CAP was defined as a discharge diagnosis code for pneumonia and an antibiotic prescription. Treatment failure was defined as an ED or primary care revisit, or admission, within 14 days during which an antibiotic change was made.

**Results:**

28 *S. pneumoniae* isolates were identified from sterile sites between 2017-2020 and 171 isolates were identified overall. All isolates from sterile sites had penicillin MICs of ≤ 2 μg/mL and 165 (96%) of isolates overall had penicillin MICs of ≤ 2 μg/mL (Table 1). Of these, 10 isolates had MICs of 2 μg/mL, all from non-sterile sites. In 2019, 589 patients were treated for CAP in the ED; 447 (76%) received amoxicillin and 142 (24%) were treated with alternative antibiotics. Treatment failures occurred in 15 amoxicillin-treated patients (3.3%, 95% confidence interval 1.9-5.5%) and in 5 patients (3.5%, 95% confidence interval 1.2-8.0%) treated with alternative antibiotics.

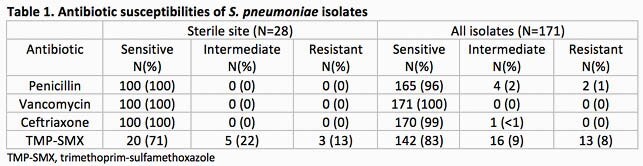

**Conclusion:**

*In vitro* penicillin resistance was rare at our institution. Further, given that *S. pneumoniae* is rarely identified by culture, we also demonstrated that clinical amoxicillin treatment failures were infrequent using twice daily amoxicillin dosing. Coupled with provider and family preference, these data supported continuing our current practice of twice daily amoxicillin dosing.

**Disclosures:**

**All Authors**: No reported disclosures

